# Period-like polynomials for *L*-series associated with half-integral weight cusp forms

**DOI:** 10.1007/s40687-024-00455-w

**Published:** 2024-06-15

**Authors:** James Branch, Nikolaos Diamantis, Wissam Raji, Larry Rolen

**Affiliations:** 1 University of Nottingham, Nottingham, UK; 2 American University of Beirut and Number Theory Research Unit at Center for Advanced Mathematical Sciences, Beirut, Lebanon; 3 Vanderbilt University, Nashville, USA

## Abstract

Given the *L*-series of a half-integral weight cusp form, we construct polynomials behaving similarly to the classical period polynomial of an integral weight cusp form. We also define a lift of half-integral weight cusp forms to integral weight cusp forms that are compatible with the *L*-series of the respective forms.

## Introduction

The Dirichlet series associated by Shimura to half-integral weight modular forms in the last section of his original paper [7] has not received as much attention as its integral weight counterpart. Partly because of its failure to possess an Euler product, it has not been extensively studied from arithmetic and algebraic perspectives that have a long history in the case of integral weight modular forms.

In view of this, the two main purposes of this note are: (i) to attach Eichler integrals and period-like polynomials to the *L*-series of a half-integral weight cusp form. This will lead to cohomology classes with coefficients in a *finite*-dimensional vector space in a way that parallels the Eichler cohomology of the integral weight case and (ii) to define a lift of half-integral weight cusp forms to integral weight cusp forms that are compatible with the *L*-series of the respective forms.

This section presents special cases of the two main results of the note. The first one is obtained from Theorem 3.1 in the special case $N=4$, *k* such that $4|(k-\frac{5}{2})$ and $a=k-2$:

### Theorem 1.1

Let $k \in \frac{1}{2}+\mathbb Z$ such that $k-\frac{5}{2} \in 4 \mathbb N$ and let *f* be a cusp form of weight *k* for $\Gamma _0(4)$ such that $f(-1/(4z))=(-2iz)^{k}f(z).$ For each *z* in the upper half-plane $\mathfrak H$ define the “Eichler integral”

$$\begin{aligned} F(z)=\Gamma (k-1)\int _z^{i \infty } f(w) \left( \sum _{n=0}^{k-\frac{5}{2}} \left( \frac{(4i)^n}{n! \Gamma (k-1-n)}+\frac{4^{k-n-\frac{11}{4}}i^{\frac{5}{2}-n} w^{-\frac{1}{2}}}{(k-\frac{5}{2}-n)!\Gamma (n+\frac{3}{2})}\right) z^nw^{k-2-n} \right) dw. \end{aligned}$$

Then,

1. For each $z \in \mathfrak H$, $P(z):=F(z)-F(-1/(4z))(-2iz)^{k-\frac{5}{2}}$ is a polynomial of degree at most $k-\frac{5}{2}$ in *z*.
2. We have
  $$\begin{aligned} P(z) =i^{k-1}\Gamma (k-1)\sum _{n=0}^{k-\frac{5}{2}}\left( \frac{4^n \Lambda _f(k-1-n)}{n! \Gamma (k-1-n)} + (-1)^{n+1} \frac{4^{k-n-\frac{11}{4}} \Lambda _f(k-\frac{3}{2}-n)}{(k-\frac{5}{2}-n)!\Gamma (n+\frac{3}{2})} \right) z^n, \end{aligned}$$
  where $\Lambda _f(s)$ is the *L*-series of *f* (to be defined precisely in the next section).

The polynomial *P* shares some of the defining features of the *period polynomial* of integral weight forms: it encodes certain values of $\Lambda _f$ inside the interval $[1, k-1]$ and satisfies one of the period relations. Indeed, in Prop. 3.2 we will show that *P* matches exactly the $(k-\frac{3}{2})$-th partial sum in the Taylor expansion of the (“symmetrised” version of the) Eichler cocycle, as extended to all real weights in [1]. Since then the period polynomial equals the value at the Fricke involution of an Eichler cocycle (based at $i \infty$), we think of *P* as an analogue of the period polynomial for half-integral weight cusp forms. The period relation of *P* (see Th. 3.1 (i)) is not immediately visible from its expression in part (2) of Th. 1.1, but it is deduced from its relation with the analogue of the Eichler integral *F*(*z*), as is the case with the classical period polynomial.

A first, to our knowledge, attempt to develop a cohomology for *L*-series of half-integral cusp forms was made in [3]. Its main construction encodes *L*-values inside $[1, k-1]$ and satisfies one of the period relations too. However, the analogue of *P* in [3] belongs to an infinite-dimensional space, whereas *P* is a polynomial of degree $\le k-\frac{5}{2}.$ Another difference is that, as will be seen in the general form of the theorem in the sequel, our polynomial *P* can be made to encode values of $\Lambda _f(s)$ at a larger class of finite “arithmetic sequences” inside $[1, k-1]$.

The second main result presented here is a special case of Theorem 4.1:

### Theorem 1.2

Let $k \in \frac{1}{2}+\mathbb Z$ such that $k-\frac{5}{2} \in 4 \mathbb N$. For each cusp form *f* of weight *k* for $\Gamma _0(4)$ such that $f(-1/(4z))=(-2iz)^{k}f(z)$ there exists a unique pair (*g*, *h*) of cusp forms of (integral) weight $k-\frac{1}{2}$ and level 4 such that, for each $n=0, \dots , k-\frac{5}{2}$, we have

1.1 $$\begin{aligned}  &   \left( \frac{2^{2n} }{n! \Gamma (k-1-n)} + (-1)^{n+1} \frac{2^{2k-5-2n} }{(k-\frac{5}{2}-n)!\Gamma (n+\frac{3}{2})} \right) \Lambda _f \left( k-\frac{5}{4}-n \right) \nonumber \\  &   \quad = \frac{i^{\frac{3}{4}}}{\Gamma (k-1)}\left( {\begin{array}{c}k-\frac{5}{2}\\ n\end{array}}\right) \left( i^{n+1}\Lambda _g \left( k-n-\frac{3}{2} \right) +i^{-n-1} \overline{\Lambda _{h}\left( k-n-\frac{3}{2}\right) }\right) \end{aligned}$$

The main characteristic of the “lift” induced by Theorem 1.2 is that it is compatible, in the sense of Eq. (1.1), with the *L*-series of the half-integral weight form and that of the corresponding integral weight forms. On the other hand, there does not seem to be any compatibility with the Hecke action, and the “lifted” forms are not explicitly given in terms of Fourier expansions, as was the case of the Shimura lift. One also notices that the weight of our “lift” is half the weight of the Shimura lift. Because of those differences in their behaviour and the different problems they were each designed to resolve, it does not seem likely that our lift is related to the Shimura lift.

The lack of compatibility with the Hecke action was expected: The identity of Theorem 1.2 expresses the “critical” values of *L*-series of a half-integral weight cusp form directly in terms of *L*-values of *integral* weight forms. If our lift were compatible with the Hecke action, then we could immediately deduce algebraicity results about the *L*-values of some half-integral weight Hecke eigenforms from the corresponding results in the integral weight case. However, algebraic properties so similar to those of the integral-weight *L*-values are not expected for *L*-series of half-integral weight forms. Therefore, additional input is required to derive algebraic information from the translation of half-integral weight *L*-values to integral weight *L*-values provided by Theorem 1.2.

It was also mentioned above that our lift is not given explicitly via Fourier expansions. Nevertheless, it does have an explicit expression through the explicit inverse of the Eichler-Shimura map given in [6]. Theorem 5.4 allows us to obtain the integral weight lift of a given form *f* of half-integral weight *k* from $k-3/2$ values of the *L*-series associated with *f*. To formulate and prove this result we develop, in Sect. 5, a reformulation of our lift of Theorem 4.1 which may be of independent interest. This reformulation (Prop. 5.2) applies only to *odd* values of *n* and relies on a version of the Eichler-Shimura isomorphism (Th. 5.1) which considers separately the even and the odd part of a polynomial. As a result, it makes it possible to express those *L*-values of *f* that correspond to odd *n* in terms of a single integral weight form. By contrast, Th. 4.1 requires two of them, but accounts for *L*-values responding to both odd and even *n*.

## Terminology and notation

We first fix the terminology and the notation we will be using. They will mostly be consistent with those of [7] and [3].

Let $k \in \frac{1}{2}+\mathbb Z$ and $N \in 4 \mathbb N.$ We let $\left( \frac{c}{d} \right)$ be the Kronecker symbol. For an odd integer *d*, we set

2.1 $$\begin{aligned} \epsilon _d:={\left\{ \begin{array}{ll} 1 &  \text { if } d\equiv 1\bmod {4}, \\ i &  \text { if } d\equiv 3\bmod {4}, \end{array}\right. } \end{aligned}$$

so that $\epsilon _d^2 = \left( \frac{-1}{d}\right)$. We set the implied logarithm to equal its principal branch so that $-\pi<$arg$(z) \le \pi$. We define the action $|_k$ of $\Gamma _0(N)$ on smooth functions *f* on $\mathfrak {H}$ as follows:

2.2 $$\begin{aligned} (f|_k\gamma )(z):= \left( \frac{c}{d} \right) \epsilon _d^{2k} (cz+d)^{-k} f(\gamma z) \qquad \text { for all } \gamma =\begin{pmatrix} * &  * \\ c &  d \end{pmatrix} \in \Gamma _0(N). \end{aligned}$$

Further, let $$W_N = \begin{pmatrix} 0& -1/\sqrt{N}\\ \sqrt{N} &  0\end{pmatrix}$$ and $\Gamma _0(N)^* = \langle W_N,\Gamma _0(N)\rangle .$ We set

2.3 $$\begin{aligned} (f|_kW_N)(z):= (-i\sqrt{N} z)^{-k} f(-1/(Nz)). \end{aligned}$$

We extend the action to $\mathbb C[\Gamma _0(N)^* ]$ by linearity.

For $n \in \mathbb Z$ we let, as usual,

2.4 $$\begin{aligned} (f|_n\gamma )(z):= (cz+d)^{-n} f(\gamma z) \qquad \text { for all } \gamma =\begin{pmatrix} * &  * \\ c &  d \end{pmatrix} \in {{\,\textrm{SL}\,}}_2(\mathbb R). \end{aligned}$$

Let $k \in \frac{1}{2}\mathbb Z$. If $\Gamma$ is either a subgroup of finite index in ${{\,\textrm{SL}\,}}_2(\mathbb Z)$ or $\Gamma ^*_0(N)$ for some *N*, and $\chi$ a character on $\Gamma$, we set

2.5 $$\begin{aligned} f|_{k, \chi }\gamma := \overline{\chi (\gamma )}f|_k \gamma \qquad \text { for all } \gamma \in \Gamma . \end{aligned}$$

We will denote the space of cusp forms of weight *k* and character $\chi$ for $\Gamma$ by $S_k(\Gamma , \chi )$. If $\chi$ is the trivial character, we write $S_k(\Gamma )$.

For $f, g \in S_k(\Gamma , \chi )$, we define the Petersson scalar product as

$$\begin{aligned} (f, g)=\int _{\Gamma \backslash \mathfrak H} f(z)\overline{g(z)}y^k \frac{dxdy}{y^2} \quad \text{ where } \, \, z=x+iy. \end{aligned}$$

Let $\lambda$ be the width of the cusp $\infty$. For integral or half-integral weights *k*, we attach to

$$\begin{aligned} f(z) = \sum _{n\ge 1}a_f(n)e^{\frac{2 \pi i nz}{\lambda }} \in S_k(\Gamma , \chi ) \end{aligned}$$

the *L*-*series*

$$\begin{aligned} L_f(s)=\sum _{n\ge 1} \frac{a_f(n)}{n^s}. \end{aligned}$$

This is absolutely convergent for $\Re (s) \gg 1$ and can be analytically continued to the entire complex plane. Its “completed” version is

2.6 $$\begin{aligned} \Lambda _f(s) = \frac{\Gamma (s) \lambda ^s}{(2\pi )^s}\sum _{n\ge 1} \frac{a_f(n)}{n^s} = \int _0^\infty f(it)t^s\frac{dt}{t}. \end{aligned}$$

For $\chi$ trivial, it satisfies the functional equation

2.7 $$\begin{aligned} \Lambda _f(s)=N^{\frac{k}{2}-s}\Lambda _{f|_kW_N}(k-s), \,\, \, \, \text {if}\,k \in \frac{1}{2}+\mathbb Z\hbox { and} \, \, \Lambda _f(s)=i^k N^{\frac{k}{2}-s}\Lambda _{f|_kW_N}(k-s), \,\, \, \text {if}\,k \in \mathbb Z. \end{aligned}$$

Finally, we fix the following notation: We set

$$\begin{aligned} T: = \begin{pmatrix} 1& 1\\ 0 &  1\end{pmatrix}, \qquad S:= \begin{pmatrix} 0& -1\\ 1 &  0\end{pmatrix}, \qquad U:=TS= \begin{pmatrix} 1& -1 \\ 1 &  0\end{pmatrix}. \end{aligned}$$

For *z*, *w* not necessarily non-negative integers, set

$$\begin{aligned} \left( {\begin{array}{c}z\\ w\end{array}}\right) :=\frac{\Gamma (z+1)}{\Gamma (w+1)\Gamma (z-w+1)}. \end{aligned}$$

## An analogue of the period polynomial

Let *f* be a cusp form of weight $k \in \frac{1}{2}+\mathbb Z$ for $\Gamma _0(N)$ such that $f|_kW_N=f$. Fix $a\in [0,2k-9/2]$ and set

$$\begin{aligned} P_a(z):= \int _0^{i\infty } f(w)\Phi _a(z,w)dw, \end{aligned}$$

where

$$\begin{aligned} \Phi _a(z,w) :=\sum _{n=0}^{k-5/2} \left[ \left( {\begin{array}{c}k-2\\ n\end{array}}\right) (iNz)^nw^{a-n}+\frac{i^k}{\root 4 \of {N}}\left( {\begin{array}{c}k-2\\ n+1/2\end{array}}\right) (iz)^n(-Nw)^{2k-9/2-a-n}\right] . \end{aligned}$$

### Theorem 3.1

Let $k \in \frac{1}{2}+\mathbb Z$ with $k>5/2$. Suppose that $f\in S_k(\Gamma ^*_0(N))$ and $a\in [0,2k-9/2]$. With the above notation, set

$$\begin{aligned} F_a(z):= \int _z^{i\infty } f(w)\Phi _a(z,w)dw. \end{aligned}$$

(i) For $z\in \mathfrak {H}$ we have
  $$\begin{aligned} -i^{k-5/2} F_a|_{5/2-k}W_N +F_a = P_a. \end{aligned}$$
  Therefore, if $\chi$ is a character on $\Gamma ^*_0(N)$ such that $\chi (W_N)=i^{\frac{5}{2}-k}$, then
  3.1 $$\begin{aligned} P_a|_{5/2-k, \chi }(W_N+1)=0. \end{aligned}$$
  In particular, if $4|(k-5/2)$, then
  $$\begin{aligned} P_a|_{5/2-k}(W_N+1)=0. \end{aligned}$$
(ii) For each $z \in \mathbb C,$
  3.2 $$\begin{aligned} P_a(z)= &   i^{a+1}\sum _{n=0}^{k-5/2}\left[ \left( {\begin{array}{c}k-2\\ n\end{array}}\right) N^n \Lambda _f(a+1-n)\right. \nonumber \\ \qquad+ &   \left. \left( {\begin{array}{c}k-2\\ n+1/2\end{array}}\right) N^{2k-a-n-\frac{19}{4}} i^{2n-k+\frac{1}{2}} \Lambda _f(2k-7/2-a-n) \right] z^n. \end{aligned}$$

### Proof

We first show that

3.3 $$\begin{aligned} -(i\sqrt{N}z)^{k-5/2}(-i\sqrt{N}w)^{k-2}\Phi _a(W_Nz,W_Nw) = \Phi _a(z,w). \end{aligned}$$

Indeed, the left-hand side is

$$\begin{aligned} -(i\sqrt{N} z)^{k-5/2}(-i\sqrt{N}w)^{k-2}\sum _{n=0}^{k-5/2}\left[ \left( {\begin{array}{c}k-2\\ n\end{array}}\right) (iz)^{-n}(-Nw)^{n-a}\right. \\ \left. + \frac{i^k}{\root 4 \of {N}}\left( {\begin{array}{c}k-2\\ n+1/2\end{array}}\right) (iNz)^{-n} w^{n-2k+9/2+a}\right] . \end{aligned}$$

Observe that

$$\begin{aligned} \left( {\begin{array}{c}k-2\\ k-5/2-n\end{array}}\right) =\left( {\begin{array}{c}k-2\\ n+1/2\end{array}}\right) , \end{aligned}$$

so that the change of variables $n\mapsto k-5/2-n$ yields

3.4 $$\begin{aligned} -(i\sqrt{N}z)^{k-5/2}(-i\sqrt{N}w)^{k-2}\sum _{n=0}^{k-5/2} \left[ \left( {\begin{array}{c}k-2\\ n+1/2\end{array}}\right) (iz)^{n-k+5/2}(-Nw)^{k-5/2-n-a}\right. \end{aligned}$$

3.5 $$\begin{aligned} \left. + \frac{i^k}{\root 4 \of {N}}\left( {\begin{array}{c}k-2\\ n\end{array}}\right) (iNz)^{n-k+5/2}w^{-k+2-n+a}\right] . \end{aligned}$$

Since $w \in \mathfrak H$, we have, for every $t\in \mathbb {R}$, $(-w)^t = e^{-\pi i t} w^t.$ A routine computation shows

$$\begin{aligned} -(i\sqrt{N}z)^{k-5/2}(-i\sqrt{N}w)^{k-2} (iz)^{n-k+5/2}(-Nw)^{k-5/2-n-a} = \frac{i^k}{\root 4 \of {N}}(iz)^n (-Nw)^{2k-9/2-a-n} \end{aligned}$$

and similarly:

$$\begin{aligned} -(i\sqrt{N}z)^{k-5/2}(-i\sqrt{N}w)^{k-2} \frac{i^k}{\root 4 \of {N}} (iN z)^{n-k+5/2}w^{2-k-n+a}=(iNz)^n w^{a-n}. \end{aligned}$$

Therefore, (3.43.5) equals $\Phi _a(z, w)$, establishing (3.3).

With (3.3) we now have:

3.6 $$\begin{aligned} i^{k-5/2} (F_a|_{5/2-k} W_N)(z)= &   (i\sqrt{N} z)^{k-5/2}\int _{W_Nz}^{i\infty } f(w)\Phi _a(W_N z,w)dw \nonumber \\= &   (i\sqrt{N}z)^{k-5/2} \int _z^0 f(W_Nw)\Phi _a(W_Nz,W_Nw) d(W_Nw) \nonumber \\= &   -(i\sqrt{N} z)^{k-5/2} \int _z^0 f(w)(-i\sqrt{N}w)^k \Phi _a(W_Nz,W_Nw)\frac{dw}{(-i\sqrt{N}w)^2} \nonumber \\= &   \int _z^0 f(w)\Phi _a(z,w)dw=F_a(z)-P_a(z). \end{aligned}$$

To prove (3.1), we have from (3.6),

$$\begin{aligned} P_a|_{\frac{5}{2}-k, \chi }(1+W_N)=F_a|_{\frac{5}{2}-k, \chi }(1-W_N)|_{\frac{5}{2}-k, \chi }(1+W_N)=F_a|_{\frac{5}{2}-k, \chi }(1-W_N^2)=0. \end{aligned}$$

To show (3.2), we expand the defining expression for $P_a$ and use the integral formula for $\Lambda _f$ in (2.6).

This theorem and (2.7) show that $P_a$ can be thought of as a “period polynomial” encoding the *L*-values of *f* at $a+1,a,a-1,\cdots , a-k+7/2$. Among the various choices of *a*, the most “canonical” is $a=k-2$ because then, our “period polynomial” $P_a$ becomes entirely consistent with the Eichler cohomology attached to general weight cusp forms, as in [1]. Specifically, the Eichler cocycle on which that cohomology is based is induced by the assignment (Section 2.2 of [1]) to $f \in S_k(\Gamma )$ of the map

$$\begin{aligned} \Gamma \ni \gamma \longrightarrow \psi _{f, \gamma }^{\infty }(z):=\int _{\gamma ^{-1}i\infty }^{i \infty } f(w)(w-z)^{k-2}dw \end{aligned}$$

where $\psi _{f, \gamma }^{\infty }$ is defined on the *lower* half-plane $\bar{\mathfrak H}$. For $\gamma =W_N$, the integral giving $\psi _{f, W_N}^{\infty }\left( x \right)$ is well-defined for $x>0$ as well. We then have the following relation between our “period polynomial” $P_{k-2}$ and $\psi _{f, W_N}^{\infty }\left( x \right)$.

### Proposition 3.2

For each $x>1$, we have

$$\begin{aligned} P_{k-2}(ix)= \psi _{f, W_N}^{\infty }\left( Nx \right) -i^{1-2k}\psi _{f, W_N}^{\infty }\left( 1/x \right) (\sqrt{N} x)^{k-\frac{5}{2}}+O(x^{k-\frac{3}{2}}). \end{aligned}$$

### Proof

We first note that

3.7 $$\begin{aligned}  &   \psi _{f, W_N}^{\infty }\left( Nx \right) -i^{1-2k}\psi _{f, W_N}^{\infty }\left( 1/x \right) (\sqrt{N} x)^{k-\frac{5}{2}} \\  &   \quad = i^{k-1}\int _0^{\infty } f(it) \left( (1+iNxt^{-1})^{k-2} -i^{1-2k}(1+ix^{-1}t^{-1})^{k-2} (\sqrt{N} x)^{k-\frac{5}{2}}\right) t^{k-2}dt.\nonumber \end{aligned}$$

By Taylor’s formula we have, for each $M \in \mathbb N$,

$$\begin{aligned}  &   (1+iNxt^{-1})^{k-2}\\  &   \quad =\sum _{n=0}^{M-1} \left( {\begin{array}{c}k-2\\ n\end{array}}\right) (iNxt^{-1})^n+O_M\left( \int _0^1(1-y)^{M-1}(1+iNyxt^{-1})^{k-2-M} (iNxt^{-1})^M dy\right) . \end{aligned}$$

If $M>k-2$ the error term is $O_M((xt^{-1})^M)$. Likewise, again if $M>k-2$, the error term of $(1+ix^{-1}t^{-1})^{k-2}$ is $O_M((x^{-1}t^{-1})^M)$. Therefore for $M=k-\frac{3}{2}$, we deduce, for $x>1$,

3.8 $$\begin{aligned}  &   \psi _{f, W_N}^{\infty }\left( Nx \right) -i^{1-2k}\psi _{f, W_N}^{\infty }\left( 1/x \right) (\sqrt{N} x)^{k-\frac{5}{2}} \nonumber \\  &   \quad = i^{k-1}\sum _{n=0}^{k-\frac{5}{2}} \left( {\begin{array}{c}k-2\\ n\end{array}}\right) \left( (iNx)^n-i^{1-2k} (ix^{-1})^n (\sqrt{N} x)^{k-\frac{5}{2}} \right) \Lambda _f(k-1-n)+O(x^{k-\frac{3}{2}})\nonumber \\ \end{aligned}$$

where the implied constant is independent of *x*. The change of variables $n \rightarrow k-\frac{5}{2}-n$, followed by (2.7) in the second sum, implies the result. $\square$

### Comparison with the period function of [3]

Another function encoding special values of $L_f$ is given in [3]. The result in [3] is stated for cusp forms on Hecke groups, but, thanks to the embedding of $S_k(\Gamma ^*_0(N))$ into a space of cusp forms for Hecke groups ((8.1) of [3]), it can be formulated for the modular forms studied in Theorem 3.1:

#### Proposition 3.3

[3] Let $k \in \frac{1}{2}+\mathbb Z$ and $f(z)=\sum a_f(n)e^{2 \pi i n z} \in S_k(\Gamma _0(N))$ such that $f|_kW_N=f$. For each $z \in \mathfrak {H}$ set

$$\begin{aligned} \mathcal E^*_f(z)=\frac{1}{\sqrt{\pi }}\sum _{n \ge 1} \frac{a_f(n)}{n^{k-1}}\left( e^{-2\pi i n z} \Gamma \left( \frac{1}{2}, -2\pi i n z \right) -\frac{1}{\sqrt{-2\pi i n z}} \right) . \end{aligned}$$

Then, for all $z \in \mathfrak H,$

3.9 $$\begin{aligned} \left( \mathcal E^*_f|_{2-k}(1-W_N)\right) (z)=\sum _{n=0}^{k-\frac{3}{2}} \left( \frac{L_f(k-n-1)}{\Gamma (n+1)}+ \frac{L_f(k-n-\frac{1}{2})}{\Gamma (n+\frac{1}{2})}\left( \frac{2\pi z}{i}\right) ^{-\frac{1}{2}}\right) \left( \frac{2\pi z}{i}\right) ^n.\qquad \qquad \end{aligned}$$

The values of $L_f(s)$ at $k-1, \dots \frac{3}{2}$ appearing in the right-hand side of (3.9) are also encoded by $P_{k-2}$. However, the function on the right-hand side of (3.9) does not belong to a finite-dimensional space closed under the action of the group. Further, the “Eichler integral” $\mathcal E^*_f(z)$ is defined as a series. To complete the comparison of our construction with the “period function” of [3], we show how the main piece of $\mathcal E^*_f(z)$ can nevertheless be expressed as an integral too.

#### Proposition 3.4

With the notation of Theorem (3.1), for each $z \in \mathfrak H$,

$$\begin{aligned} \mathcal E^*_f(z)=\alpha _k (-iz)^{\frac{1}{2}} \int _{z}^{i \infty } F_f(w) (w-z)^{k-\frac{5}{2}} dw-\frac{1}{\sqrt{-2 \pi ^2 i z}}L_f\left( k-\frac{1}{2}\right) \end{aligned}$$

where

$$\begin{aligned} F_f(w):= \int _0^{\infty } f\left( xw\right) x^{k-\frac{3}{2}}(x+1)^{-\frac{1}{2}} dx \end{aligned}$$

and $$\alpha _k=(-2 \pi i)^{k-1}/ (\pi ^{\frac{1}{2}}(k-\frac{5}{2})!).$$

#### Proof

We first recall ([5], (8.19.1), (8.19.3)) that, for Re$(w)>0,$

$$\begin{aligned} \Gamma \left( \frac{1}{2}, w \right) =w^{\frac{1}{2}}\int _1^{\infty }e^{-wt} t^{-\frac{1}{2}} dt. \end{aligned}$$

Therefore, for $$z \in \mathfrak H,$$

$$\begin{aligned}  &   \sum _{n \ge 1} \frac{a_f(n)}{n^{k-1}}\frac{e^{-2\pi i n z }}{\sqrt{\pi }} \Gamma \left( \frac{1}{2}, -2\pi i n z \right) = \frac{1}{\sqrt{\pi }} \sum _{n \ge 1} \frac{a_f(n)}{n^{k-1}} e^{-2\pi i n z } \left( -2\pi i n z \right) ^{\frac{1}{2}}\int _1^{\infty } e^{2\pi i n zt }t^{-\frac{1}{2}} dt\\  &   \quad =(-2 i z)^{\frac{1}{2}} \int _1^{\infty }t^{-\frac{1}{2}} \left( \sum _{n \ge 1} \frac{a_f(n)}{n^{k-\frac{3}{2}}} e^{2 \pi i n z(t-1)} \right) dt. \end{aligned}$$

By the theory of usual (integral weight) Eichler integrals, followed by the changes of variables $x=t-1$ and $w_1=w/x$, this equals

$$\begin{aligned}  &   (-2 i z)^{\frac{1}{2}} \int _1^{\infty }t^{-\frac{1}{2}} \frac{(-2 \pi i)^{k-\frac{3}{2}}}{\Gamma (k-\frac{3}{2})} \int _{z(t-1)}^{i \infty } f(w) \left( w-z(t-1)\right) ^{k-\frac{5}{2}} dwdt\\  &   \quad = \alpha _k z^{\frac{1}{2}} \int _0^{\infty }x^{k-\frac{3}{2}} (x+1)^{-\frac{1}{2}} \int _{z}^{i \infty } f(xw_1)\left( w_1-z \right) ^{k-\frac{5}{2}} dw_1 dx. \end{aligned}$$

A change in the order of integration implies the formula.

## An Eichler cocycle

In this section, we will first use Theorem 3.1 to construct an Eichler cocycle, with coefficients in the space $\mathbb {C}_{k-5/2}[z]$. We maintain the notation and assumptions of the last section.

Since 4|*N*, $\Gamma _0(N)/\{\pm 1\}$ is torsion-free and hence free on a set of generators $\{\gamma _j\}_{j=1}^{2g+h-1}$, where $\gamma _1=T$, *g* is the genus and *h* the number of inequivalent cusps of $\Gamma _0(N)$ ([2], Prop. 2.4; here by $T, \gamma _j$, etc. we mean the images of those elements of $\Gamma _0(N)$ into $\Gamma _0(N)/\{\pm 1\}$). We also have $W_N\Gamma _0(N)=\Gamma _0(N)W_N$ and thus $\Gamma ^*_0(N)=\langle \Gamma _0(N), W_N \rangle$ is generated by $\{\gamma _j\} \cup \{W_N\}$ with only relations $(-1)^2=1$ and $W_N^4=1$.

From the above, we first deduce that there is always a character $\chi$ on $\Gamma ^*_0(N)$ such as prescribed in Theorem 3.1, that is such that $\chi (W_N)=i^{5/2-k}$. Indeed, since $i^{4(5/2-k)}=1$, the character induced by the assignment $\chi (-1)=(-1)^{5/2-k}$, $\chi (W_N)=i^{5/2-k}$ and $\chi (\gamma _i)=1$ for $i=1, \dots 2\,g+h-1$ is well-defined.

Further, let, for $a \in [0, 2k-\frac{9}{2}]$, $P_a$ be the polynomial defined in (3.2). Consider the polynomial

$$\begin{aligned} \hat{P}_a(z):=P_a(2z/\sqrt{N}). \end{aligned}$$

It is then easy to deduce from Theorem 3.1 that

4.1 $$\begin{aligned} \hat{P}_a|_{\frac{5}{2}-k, \chi }(W_4+1)=0 \end{aligned}$$

for any character $\chi$ on $\Gamma ^*_0(4)$ such that $\chi (W_4)=i^{\frac{5}{2}-k}$. (Since there is no risk of confusion, we use the same notation for both characters). Recall that $\Gamma _0(4)/\{\pm 1\}$ is freely generated on the generators

$$\begin{aligned} T, \begin{pmatrix} 1 &  0 \\ 4 &  1 \end{pmatrix} \end{aligned}$$

and thus $\Gamma ^*_0(4)$ is generated by $-1, T, W_4$ in $\Gamma ^*_0(4)$ with only relations $(-1)^2=1$ and $W_4^4=1$. We consider the map

$$\begin{aligned} \hat{\pi }_f: \Gamma ^*_0(4) \rightarrow \mathbb {C}_{k-5/2}[z] \end{aligned}$$

induced by the 1-cocycle condition from the values

$$\begin{aligned} \hat{\pi }_f(W_4) = \hat{P}_a \qquad \text{ and } \qquad \hat{\pi }_f(-1)=\hat{\pi }_f(T)=0. \end{aligned}$$

Then $\hat{\pi }_f$ is well-defined since by (4.1),

$$\begin{aligned} \hat{\pi }_f(W_4^2) = \hat{\pi }_f(W_4)|_{\frac{5}{2}-k, \chi } W_4 +\hat{\pi }_f(W_4) = \hat{P}_a|_{\frac{5}{2}-k}W_4 + \hat{P}_a = 0. \end{aligned}$$

This cocycle induces a non-trivial class in $H_{\textrm{par}}^{1}(\Gamma ^*_0(4), \mathbb {C}_{k-5/2}[z])$ where the action of $\Gamma ^*_0(4)$ on $\mathbb {C}_{k-5/2}[z]$ is $|_{5/2-k, \chi }$.

On the other hand, according to the Eichler-Shimura isomorphism, there is an isomorphism

$$\begin{aligned} \phi : S_{k-{1/2}}(\Gamma ^*_0(4), \chi ) \oplus \overline{S_{k-{1/2}}(\Gamma ^*_0(4), \bar{\chi })} \longrightarrow H_{\textrm{par}}^{1}(\Gamma ^*_0(4), \mathbb {C}_{k-5/2}[z]) \end{aligned}$$

induced by the assignment of the following map $\phi (g, \bar{h}):\Gamma ^*_0(4) \rightarrow \mathbb {C}_{k-5/2}[z]$ to $(g, \bar{h})$:

$$\begin{aligned} \phi (g, \bar{h})(\gamma )= \int _{\infty }^{\gamma ^{-1} \infty } g(w)(w-z)^{k-{5/2}}dw+\int _{\infty }^{\gamma ^{-1} \infty } \overline{h(w)}(\bar{w}-z)^{k-{5/2}}d \bar{w}. \end{aligned}$$

Therefore, there are unique $g \in S_{k-1/2}(\Gamma ^*_0(4), \chi )$, $h \in S_{k-1/2}(\Gamma ^*_0(4), \bar{\chi })$ and a polynomial *Q* in $\mathbb {C}_{k-5/2}[z]$ such that

$$\begin{aligned} \hat{\pi }_f(\gamma )=\phi (g, \bar{h})(\gamma )+Q|_{\frac{5}{2}-k, \chi }(\gamma -1) \end{aligned}$$

for all $\gamma \in \Gamma ^*_0(4)$. Since $\hat{\pi }_f(T)=\phi (g, \bar{h})(T)=0$, the polynomial *Q* should vanish too, because it would otherwise have infinitely many zeros. Therefore, for each $f \in S_k(\Gamma ^*_0(4), \chi ),$ there are unique $g \in S_{k-{1/2}}(\Gamma ^*_0(4), \chi )$, $h \in S_{k-{1/2}}(\Gamma ^*_0(4), \bar{\chi })$ such that, for all $\gamma \in \Gamma ^*_0(4),$

4.2 $$\begin{aligned} \hat{\pi }_f(\gamma )=\phi (g, \bar{h})(\gamma ). \end{aligned}$$

An application of the binomial theorem combined with the integral form of $\Lambda _g(s), \Lambda _h(s)$ implies that $\phi (g, \bar{h})(W_4)$ can be expressed as a polynomial with coefficients involving values of the critical values of $g, \bar{h}$. In our case this gives

4.3 $$\begin{aligned}  &   \phi (g, \bar{h})(W_4) \\  &   \quad =- \sum _{n=0}^{k-5/2}\left( {\begin{array}{c}k-5/2\\ n\end{array}}\right) (-z)^n\left( i^{k-n-3/2}\Lambda _g(k-n-3/2)+i^{3/2+n-k} \overline{\Lambda _{h}(k-n-3/2)}\right) .\nonumber \end{aligned}$$

Comparing coefficients with the expression for $\hat{\pi }_f(W_4)=\hat{P}_a$ in (3.2), we deduce

### Theorem 4.1

Let $k \in \frac{1}{2}+\mathbb Z$ with $k>\frac{5}{2}.$ For each cusp form *f* of weight *k* for $\Gamma _0(N)$ such that $f(-1/(Nz))=(-i\sqrt{N}z)^{k}f(z)$, a character $\chi$ on $\Gamma _0^*(4)$ such that $\chi (W_4)=i^{\frac{5}{2}-k}$ and for each $a \in [0, 2k-\frac{9}{2}]$, there exists a unique pair (*g*, *h*) of cusp forms of (integral) weight $k-\frac{1}{2}$, level 4 and character $\chi$ such that, for each $n=0, \dots , k-\frac{5}{2}$, we have

4.4 $$\begin{aligned}  &   i^{a+1}2^n\left( \left( {\begin{array}{c}k-2\\ n\end{array}}\right) N^{\frac{n}{2}} \Lambda _f(a+1-n)\right. \nonumber \\  &   \left. \quad + i^{2n+\frac{1}{2}-k} \left( {\begin{array}{c}k-2\\ n+\frac{1}{2}\end{array}}\right) N^{2k-a-\frac{3n}{2}-\frac{19}{4}}\Lambda _f(2k-\frac{7}{2}-a-n) \right) \nonumber \\  &   \quad = \left( {\begin{array}{c}k-\frac{5}{2}\\ n\end{array}}\right) \left( i^{n+\frac{1}{2}+k}\Lambda _g(k-n-\frac{3}{2})+i^{-n-\frac{1}{2}-k} \overline{\Lambda _{h}(k-n-\frac{3}{2})}\right) . \end{aligned}$$

Theorem 1.2 is a special case of this for $N=4$, *k* such that $4|(k-\frac{5}{2})$ and $a=k-\frac{9}{4}$.

### A special case

In low dimensions, Theorem 4.1 can assume a simpler form. For example, we can specialise to *k* such that $\textrm{dim} S_{k-1/2}(\Gamma _0^*(4)) =1$ and $a=k-9/4$. Then, if *g* is a normalised eigenform in $S_{k-1/2}(\Gamma _0^*(4))$, which, in particular, implies that *g* has real Fourier coefficients at infinity, Theorem 4.1 becomes

#### Corollary 4.2

Let $k \in \frac{5}{2}+4\mathbb N$ such that $S_{k-1/2}(\Gamma _0^*(4))$ is 1-dimensional, spanned by a normalised cusp form *g*. For each cusp form *f* of weight *k* for $\Gamma _0(N)$ such that $f(-1/(Nz))=(-i\sqrt{N}z)^{k}f(z)$ there exists a $\lambda _f \in \mathbb C$ such that, for each $n=0, \dots , k-\frac{5}{2}$, we have

4.5 $$\begin{aligned} \Lambda _f \left( k-\frac{5}{4}-n \right) = C_{k,N,n} \Lambda _g \left( k-\frac{3}{2}-n \right) , \end{aligned}$$

where

$$\begin{aligned} C_{k,N,n} = \left( {\begin{array}{c}k-\frac{5}{2}\\ n\end{array}}\right) 2^{-n}\left( i^{\frac{7}{4}+n}+\lambda _f i^{-n-\frac{1}{4}}\right) \left[ \left( {\begin{array}{c}k-2\\ n\end{array}}\right) N^{\frac{n}{2}} + (-1)^{n+1}\left( {\begin{array}{c}k-2\\ n+\frac{1}{2}\end{array}}\right) N^{k-\frac{5}{2}-\frac{3n}{2}}\right] ^{-1}. \end{aligned}$$

**Remark.** In view of the corollary, it is tempting to try to deduce algebraicity dependence for *L*-values of *f* from the corresponding properties for integral weight forms (e.g. via Manin’s periods’ theorem). However, the number of *L*-values of *f* whose algebraic dependence we would like to derive is the same as the number of independent *L*-values of *g* in the RHS of (4.5). In the case of $k=13/2$ for instance, the two values for odd $n \in \{0, \dots , 4\}$ are essentially the same because of the functional equation of $\Lambda _f(s)$, leaving us with a single value of $\Lambda _f(s)$, trivially accounted for by the single constant $\lambda _f$.

## An explicit form of the lift

The lift described in Theorem 4.1 can be made explicit via the explicit inverse of the Eichler-Shimura map of [6]. To this end, we first reformulate the construction leading to Theorem 4.1 in a way that parallels the decomposition of the classical period polynomial into an “even” and “odd” part. For simplicity, in this section, we will be working with weight *k* such that $4|(k-\frac{5}{2}).$

We will first review some cohomological constructions used in [6] and some notation from [4] which we will then apply to half-integral weight cusp forms.

We first consider the Hecke group *H*(2) generated by the images of $T^2$ and *S* under the natural projection of ${{\,\textrm{SL}\,}}_2(\mathbb Z)$ onto PSL$_2(\mathbb Z)$. Since $4|(k-\frac{5}{2})$, it will be legitimate to use the same notation for elements of ${{\,\textrm{SL}\,}}_2(\mathbb Z)$ and their images in PSL$_2(\mathbb Z)$. The group *H*(2) has only the relation $S^2=1$ (see Sect. 5 of [3] for the summary of some basic properties of the Hecke groups).

We now recall a construction of [4] that is used to provide an explicit formula for cocycles on ${{\,\textrm{PSL}\,}}_2(\mathbb Z)$ induced by cocycles on *H*(2). It is easy to see that a set of representatives of $H(2) \backslash {{\,\textrm{PSL}\,}}_2(\mathbb Z)$ is

$$\begin{aligned} \{1, T, U\}, \qquad \text{ where } \, \, U=TS. \end{aligned}$$

To be able to keep track of coset representatives, we denote by $u:{{\,\textrm{PSL}\,}}_2(\mathbb Z)\rightarrow \{1,T,U\}$ the map which sends *x* to its corresponding coset representative in $H(2)\backslash {{\,\textrm{PSL}\,}}_2(\mathbb Z)$. Specifically, let $x \in {{\,\textrm{PSL}\,}}_2(\mathbb Z)$. If $H(2)x=H(2)$ (resp. *H*(2)*T*, *H*(2)*U*), set $u(x)=1$, (resp. *T*, *U*). Some values of *u* that will be repeatedly used below tacitly are: $u(T^{-1})=T, u(TST^{-1})=U.$

For each $x, g \in {{\,\textrm{PSL}\,}}_2(\mathbb Z)$, set

5.1 $$\begin{aligned} \kappa _{x, g}:=u(x)gu(xg)^{-1} \in H(2). \end{aligned}$$

One notices that, if $x \in H(2)$, $\kappa _{x, g}=gu(g)^{-1}$ and, if $x, g \in H(2)$, $\kappa _{x, g}=g$. Further, if $H(2)x=H(2)x'$, then $\kappa _{x, g}=\kappa _{x', g}$ and thus $\kappa _{x, g}$ is well-defined as a function of $\left( H(2) \backslash {{\,\textrm{PSL}\,}}_2(\mathbb Z) \right) \times {{\,\textrm{PSL}\,}}_2(\mathbb Z).$ Finally, $\kappa$ satisfies the relation

5.2 $$\begin{aligned} \kappa _{x, g_1g_2}=\kappa _{x, g_1}\kappa _{xg_1, g_2}. \end{aligned}$$

Next, we consider the space

$$\begin{aligned} I_k:=\text {Ind}^{\text {PSL}_2(\mathbb Z)}_{H(2)}(\mathbb {C}_{k-5/2}[z])=\{f: H(2) \backslash {{\,\textrm{PSL}\,}}_2(\mathbb Z) \rightarrow \mathbb {C}_{k-5/2}[z]\}. \end{aligned}$$

Since ${{\,\textrm{PSL}\,}}_2(\mathbb Z)$ acts on $\mathbb {C}_{k-5/2}[z]$, there is an action of ${{\,\textrm{PSL}\,}}_2(\mathbb Z)$ on $I_k$, given, for $v \in I_k$, by

$$\begin{aligned} (v||g)(x):=v(xg^{-1})|_{\frac{5}{2}-k}g \quad \text{ for } \text{ all } \, \, x \in H(2) \backslash {{\,\textrm{PSL}\,}}_2(\mathbb Z),\, g \in {{\,\textrm{PSL}\,}}_2(\mathbb Z). \end{aligned}$$

Let $\sigma : H(2) \rightarrow I_k$ be a 1-cocycle with values in $I_k$. Then, as in the case of the classical period polynomial, one sees directly by the cocycle relation that $\sigma (U)$ belongs to the space

$$\begin{aligned} W:=\{v \in I_k; v||(S+1)=v||(U^2+U+1)=0\} \end{aligned}$$

called the space of *period polynomials* in [6]. (Note that the condition $v||(-1)=v$ included in the definition of that space in [6] is not needed here because $k-\frac{5}{2}$ is even.) We also define the following subspace of *W*:

$$\begin{aligned} C:=\{P||(1-S); P \in I_k, P||T=P\}. \end{aligned}$$

As in the classical case again, the spaces *W* and *C* can be decomposed as a direct sum of the ±-eigenspaces of a certain involution. Specifically, let $$\epsilon =\left( {\begin{matrix} -1&  0 \\ 0 & 1 \end{matrix}}\right) $$ and let $\epsilon$ act on a $v \in I_k$ so that

$$\begin{aligned} (v||\epsilon )(x):=v(\epsilon x \epsilon )|_{\frac{5}{2}-k} \epsilon \quad \text{ for } \text{ all } \, \, x \in H(2) \backslash {{\,\textrm{PSL}\,}}_2(\mathbb Z). \end{aligned}$$

Then $I_k$ decomposes into ±-eigenspaces under the action of $\epsilon$, denoted $I_k^{\pm }$. Further, since *W* (resp. *C*) is closed under the action of $\epsilon$, it also decomposes into ±-eigenspaces $W^{\pm }$ (resp. $C^{\pm }$). We denote that ±-component of $\sigma (U)$ by $\sigma (U)^{\pm }$, i.e.

$$\begin{aligned} \sigma (U)^{\pm }=\frac{1}{2}\left( \sigma (U) \pm \sigma (U)||\epsilon \right) \in W^{\pm }. \end{aligned}$$

The Eichler-Shimura theorem can be formulated as a pair of isomorphisms to (quotients of) $W^{\pm }$. We present it here in the arrangement of [6] (Theorem 2.1) as applied to our setting. First, for each cusp form *g* of weight $k-\frac{1}{2}$ for *H*(2), we let $\rho _g$ be the element of $I_k$ defined by

5.3 $$\begin{aligned} \rho _g(\gamma )(z)=\int _0^{i\infty } \left( g \Big |_{k-\frac{1}{2}} \gamma \right) (w)(w-z)^{k-\frac{5}{2}}dw \quad \text{ for } \text{ all } \, \, \gamma \in H(2) \backslash {{\,\textrm{PSL}\,}}_2(\mathbb Z). \end{aligned}$$

Each polynomial $\rho _g(\gamma )(z)$ can be expanded as

5.4 $$\begin{aligned} \rho _g(\gamma )(z) = \sum _{n=0}^{k-\frac{5}{2}}(-1)^{n}\left( {\begin{array}{c}k-\frac{5}{2}\\ n\end{array}}\right) r_{\gamma , n}(g) z^{k-\frac{5}{2}-n}, \nonumber \\ \quad \text{ for } \, \, r_{\gamma , n}(g)=i^{n+1}\int _0^{\infty }\left( g \Big |_{k-\frac{1}{2}} \gamma \right) (it)t^ndt. \end{aligned}$$

Further, it can be shown that $\rho _g \in W$ and hence that it can be written as a sum of its $+$ and − components $\rho ^{\pm }_g \in W^{\pm }$. With this notation we have

### Theorem 5.1

([6], Theorem 2.1 (Eichler-Shimura)) The assignments $g \rightarrow \rho ^+_g$ and $g \rightarrow \rho ^-_g$ induce isomorphisms

$$\begin{aligned} \rho ^{+}: S_{k-\frac{1}{2}}(H(2)) \cong W^+/C^+ \quad \text{ and } \, \, \rho ^{-}: S_{k-\frac{1}{2}}(H(2)) \cong W^-/C^-. \end{aligned}$$

Since *H*(2) is ${{\,\textrm{SL}\,}}_2(\mathbb Z)$-conjugate to $\Gamma _0(2)$, Prop. 4.4 of [6] shows that

$$\begin{aligned} C^- \simeq C_{\Gamma _0(2)}^- = \{0\}, \end{aligned}$$

where $C_{\Gamma _0(2)}^-$ denotes the analogue for $C^-$ for $\Gamma _0(2)$, instead of *H*(2).

Let $k \in \frac{1}{2}+\mathbb Z$ with $k>\frac{5}{2}$ and $4|(k-\frac{5}{2})$. We will construct an element of $W^-$ based on a cusp form *f* of weight *k* for $\Gamma _0(N)$ such that $f(-1/(Nz))=(-i\sqrt{N}z)^{k}f(z)$. In the last section, we defined the map $\hat{\pi }_f: \Gamma ^*_0(4) \rightarrow \mathbb {C}_{k-5/2}[z]$ induced by the 1-cocycle condition from the values $\hat{\pi }_f(W_4) = \hat{P}_a$ and $\hat{\pi }_f(-1)=\hat{\pi }_f(T)=0.$ Since $4|(k-\frac{5}{2})$, the 1-cocycle condition of $\hat{\pi }_f$ implies that there is a well-defined 1-cocycle $\pi '_f: H(2)\rightarrow \mathbb {C}_{k-5/2}[z]$ which is induced by the 1-cocycle relation from the values

$$\begin{aligned} \pi '_f(S)(z) = \hat{P}_a(z/2) \qquad \text{ and } \pi '_f(T^2)=0. \end{aligned}$$

This cocycle gives a non-trivial class in $H_{\textrm{par}}^{1}(H(2), \mathbb {C}_{k-5/2}[z])$ where the action of *H*(2) on $\mathbb {C}_{k-5/2}[z]$ is $|_{5/2-k}$.

We now define a 1-cocycle of ${{\,\textrm{PSL}\,}}_2(\mathbb Z)$ with coefficients in $I_k$ induced by the cocycle $\pi '_f$. For each $g \in {{\,\textrm{PSL}\,}}_2(\mathbb Z)$ let $\pi _f(g)$ be the element of $I_k$ such that

5.5 $$\begin{aligned} \pi _f(g)(x):=\pi '_f(\kappa ^{-1}_{x, g^{-1}})|_{\frac{5}{2}-k} u(x)\quad \text{ for } \text{ all } \, \, x \in H(2) \backslash {{\,\textrm{PSL}\,}}_2(\mathbb Z). \end{aligned}$$

By Shapiro’s lemma or, directly with (5.1), we can see that it is a 1-cocycle. Then, as mentioned when we introduced *W* earlier in this section, $\pi _f(U) \in W$ and $\pi _f(U)=\pi ^+_f(U)+\pi ^-_f(U)$, for

$$\begin{aligned} \pi _f(U)^{\pm }=\frac{1}{2}\left( \pi _f(U) \pm \pi _f(U)||\epsilon \right) \in W^{\pm }. \end{aligned}$$

We can now state and prove another version of our lift of half-integral weight forms.

### Proposition 5.2

Let $k \in \frac{1}{2}+\mathbb Z$ such that $k>\frac{5}{2}$ and $4|(k-\frac{5}{2})$. For each $f \in S_k(\Gamma _0(N))$ such that $f|_kW_N=f$ and for each $a \in [0, 2k-\frac{9}{2}]$, there is a $g \in S_{k-\frac{1}{2}}(\Gamma ^{*}_0(4))$ such that $g|_{k-\frac{1}{2}}W_4=g$ and, for all odd $n \in \{1, \dots , k-\frac{7}{2}\}$,

5.6 $$\begin{aligned}  &   i^{a+1}\left( \left( {\begin{array}{c}k-2\\ n\end{array}}\right) N^{\frac{n}{2}} \Lambda _f(a+1-n)+ (-1)^{n+1} \left( {\begin{array}{c}k-2\\ n+\frac{1}{2}\end{array}}\right) N^{2k-a-\frac{3n}{2}-\frac{19}{4}}\Lambda _f(2k-\frac{7}{2}-a-n) \right) \nonumber \\  &   \quad = \left( {\begin{array}{c}k-\frac{5}{2}\\ n\end{array}}\right) i^{-1-n}2^{k-\frac{3}{2}-n}\Lambda _{g}\left( k-\frac{3}{2}-n\right) . \end{aligned}$$

### Proof

By Theorem 5.1, there exists a $g_1 \in S_k(H(2))$ such that

5.7 $$\begin{aligned} \pi _f(U)^{-}=\rho ^{-}_{g_1}. \end{aligned}$$

Since $H(2)\epsilon T\epsilon =H(2)T$ and $H(2)\epsilon U\epsilon =H(2)T^{-1}S^{-1}=H(2)U$, we see that for each $x \in H(2) \backslash {{\,\textrm{PSL}\,}}_2(\mathbb Z)$, $\pi _f(U)^{-}(x)$ (resp. $\rho ^{-}_{g_1}(x)$) is the part of the polynomial $\pi _f(U)(x)$ (resp. $\rho _{g_1}(x)$) corresponding to its odd powers. By the definition of $\pi _f$ and it 1-cocycle condition,

$$\begin{aligned} \pi _f(U)(1)=\pi '_f(\kappa ^{-1}_{1, U^{-1}})=\pi '_f(T^2S)=\pi '_f(T^2)|_{\frac{5}{2}-k}S+\pi '_f(S)=0+\pi '_f(S)=P_a(z/\sqrt{N}). \end{aligned}$$

This, together with (3.2), shows that the *n*-th coefficient of $\pi _f(U)(1)$ equals the left-hand side of (5.6).

By (5.4), the *n*-th coefficient of $\rho _{g_1}(1)$ equals

$$\begin{aligned} (-1)^n\left( {\begin{array}{c}k-\frac{5}{2}\\ n\end{array}}\right) i^{1-n}\Lambda _{g_1}\left( k-\frac{3}{2}-n\right) . \end{aligned}$$

Since, by the analogue of the proposition in Section 8 of [3] for integral weights, the function *g* such that $g(z):=g_1(2z)$ is a weight $k-\frac{1}{2}$ cusp form for $\Gamma ^*_0(4)$ and $\Lambda _{g_1}(s)=2^s\Lambda _{g}(s)$, we obtain the expression in the right-hand side of (5.6). With (5.7), we deduce the result.

We will identify explicitly the integral weight cusp form to which a half-integral weight form is lifted according to Prop 5.2. We will use a theorem of [6] providing explicit inverses for the Eichler-Shimura maps $\rho ^{\pm }$. To state it, we introduce some additional notation. Firstly, for each $\gamma \in H(2)\backslash {{\,\textrm{PSL}\,}}_2(\mathbb Z)$ and $n=0, \dots k-\frac{5}{2}$, we denote by $r^{\pm }_{\gamma , n}(g)$ the constants such that

$$\begin{aligned} \rho ^{\pm }_g(\gamma )(z) = \sum _{n=0}^{k-\frac{5}{2}}(-1)^{n}\left( {\begin{array}{c}k-\frac{5}{2}\\ n\end{array}}\right) r^{\pm }_{\gamma , n}(g) z^{k-\frac{5}{2}-n}. \end{aligned}$$

In particular, $r^{+}_{\gamma , n}(g)=0$ (resp. $r^{-}_{\gamma , n}(g)=0$), when *n* is odd (resp. even). Then, for each $\gamma \in H(2)\backslash {{\,\textrm{PSL}\,}}_2(\mathbb Z)$ and $0 \le n \le k-\frac{5}{2}$ we denote by $R^{\pm }_{\gamma , n}$ the unique element of $S_{k-\frac{1}{2}}(H(2))$ such that

$$\begin{aligned} r^{\pm }_{\gamma , n}(h)=(h, R^{\pm }_{\gamma , n}) \quad \text{ for } \text{ all } \, \, h \in S_{k-\frac{1}{2}}(H(2)) \end{aligned}$$

and

$$\begin{aligned} s^{\pm }_{\gamma , n}(h) = \sum _{j=0}^n \left( {\begin{array}{c}n\\ j\end{array}}\right) (-1)^{n-j}r^{\pm }_{\gamma , j}(h). \end{aligned}$$

With this notation, Theorem 6.1 of [6] reads, in our case, as

### Theorem 5.3

[6] For each $g_1 \in S_{k-\frac{1}{2}}(H(2))$, we have

$$\begin{aligned} g_1=\frac{2}{3}(2i)^{\frac{3}{2}-k}\sum _{\gamma \in H(2)\backslash {{\,\textrm{PSL}\,}}_2(\mathbb Z)} \sum _{n=0}^{k-\frac{5}{2}} \left( {\begin{array}{c}k-\frac{5}{2}\\ n\end{array}}\right) s^{-}_{\gamma U^{-1}, n}(g_1)R^{+}_{\gamma , n}. \end{aligned}$$

To use this theorem, we first compute $\pi _f(U)(\gamma )$ for $\gamma \in H(2) \backslash {{\,\textrm{PSL}\,}}_2(\mathbb Z)$:

5.8 $$\begin{aligned}&\pi _f(U)(1)=\pi '_f(\kappa ^{-1}_{1, S^{-1}})=\pi _f'(S)=P_a(z/\sqrt{N}), \\&\pi _f(U)(T)=\pi '_f(\kappa ^{-1}_{T, U^{-1}})|_{k-\frac{5}{2}}T=\pi _f'(S^{-1})|_{k-\frac{5}{2}}T^{-1}=P_a((z-1)/\sqrt{N}), \nonumber \\&\pi _f(U)(U)=\pi '_f(\kappa ^{-1}_{U, U^{-1}})|_{k-\frac{5}{2}}U=\pi '_f(UU^{-1})|_{k-\frac{5}{2}}U=0. \nonumber \end{aligned}$$

Therefore, if $g_1$ is the weight $k-\frac{1}{2}$ cusp form for *H*(2) induced from *f* by the proof of (5.6), then (5.7) implies that, for *n* odd, the *n*-th coefficient of $\rho ^{-}_{g_1}(1)$ (resp. $\rho ^{-}_{g_1}(T)$), equals the *n*-th coefficient of $P_a(z/\sqrt{N})$ (resp $P_a((z-1)/\sqrt{N})$). Thus, with (5.7) and (3.2), we deduce that, for each $\gamma \in H(2) \backslash {{\,\textrm{PSL}\,}}_2(\mathbb Z)$ and odd *n*,

$$\begin{aligned} -\left( {\begin{array}{c}k-\frac{5}{2}\\ n\end{array}}\right) r^-_{1, n}(g_1) =\frac{\alpha _{k-\frac{5}{2}-n}}{N^{\frac{k}{2}-\frac{5}{4}-\frac{n}{2}}} \quad \text {and} \, \, \left( {\begin{array}{c}k-\frac{5}{2}\\ n\end{array}}\right) r^-_{T, n}(g_1)\\ = \sum _{j=k-\frac{5}{2}-n}^{k-\frac{5}{2}}(-1)^j\frac{\alpha _j}{N^{\frac{j}{2}}} \left( {\begin{array}{c}j\\ k-\frac{5}{2}-n\end{array}}\right) \end{aligned}$$

where

$$\begin{aligned} \alpha _j:=i^{a+1}\left( \left( {\begin{array}{c}k-2\\ n\end{array}}\right) N^n \Lambda _f(a+1-n)\right. \\ \left. +\left( {\begin{array}{c}k-2\\ n+\frac{1}{2}\end{array}}\right) N^{2k-a-n-\frac{19}{4}} i^{2n-k+\frac{1}{2}} \Lambda _f(2k-\frac{7}{2}-a-n)\right) . \end{aligned}$$

This, in turn, implies that, for each $n=0, \dots k-\frac{5}{2}$,

5.9 $$\begin{aligned} s^{-}_{1, n}:=s^{-}_{1, n}(g_1)&=(-1)^{n} \sum _{\begin{array}{c} j=0\\ j\text { odd} \end{array}}^n \left( {\begin{array}{c}n\\ j\end{array}}\right) \left( {\begin{array}{c}k-\frac{5}{2}\\ j\end{array}}\right) ^{-1} \frac{\alpha _{k-\frac{5}{2}-j}}{N^{\frac{k}{2}-\frac{5}{4}-\frac{j}{2}}} \qquad \text {and}\\ s^{-}_{T, n}:=s^{-}_{T, n}(g_1)&=(-1)^{n+1} \sum _{\begin{array}{c} j=0 \\ j\text { odd} \end{array}}^n \left( {\begin{array}{c}n\\ j\end{array}}\right) \left( {\begin{array}{c}k-\frac{5}{2}\\ j\end{array}}\right) ^{-1} \left( \sum _{\ell =k-\frac{5}{2}-j}^{k-\frac{5}{2}}(-1)^{\ell }\frac{\alpha _\ell }{N^{\frac{\ell }{2}}} \left( {\begin{array}{c}\ell \\ k-\frac{5}{2}-j\end{array}}\right) \right) \nonumber \end{aligned}$$

Recall the cusp form $g(z)=g_1(2z)$ of weight $k-\frac{1}{2}$ for $\Gamma _0^*(4)$. Then, with Theorem 5.3, we deduce

### Theorem 5.4

Let $k \in \frac{1}{2}+\mathbb Z$ such that $k>\frac{5}{2}$ and $4|(k-\frac{5}{2})$. For each $f \in S_k(\Gamma _0(N))$ such that $f|_kW_N=f$ and for each $a \in [0, 2k-\frac{9}{2}]$, the “lift” $g \in S_{k-\frac{1}{2}}(\Gamma ^{*}_0(4))$ of Prop. 5.2 is given by

$$\begin{aligned} g(z)= \frac{2}{3}(2i)^{\frac{3}{2}-k} \sum _{n=0}^{k-\frac{5}{2}} \left( {\begin{array}{c}k-\frac{5}{2}\\ n\end{array}}\right) \left( s^{-}_{1, n} R^{+}_{U, n}(2z)+ s^{-}_{T, n} R^{+}_{1, n}(2z), \right) \end{aligned}$$

where $s^{-}_{1, n}, s^{-}_{T, n}$ are given by (5.9).

## Data Availability

The authors confirm that this manuscript has no associated data.
